# Supplementary material for: Adjustment of the Structure of the Simplest Amino Acid Present in Nature—Glycine, toward More Environmentally Friendly Ionic Forms of Phenoxypropionate-Based Herbicides
Source: Int J Mol Sci. 2023 Jan 10;24(2):1360. doi: 10.3390/ijms24021360 (PMC9863448; doi:10.3390/ijms24021360)
Supplement: Supplementary file 1 [file ijms-24-01360-s001.zip › ijms-2138884-supplementary.pdf]

ELECTRONIC SUPPORTING INFORMATION

Adjustment of the Structure of the Simplest Amino Acid Present in Nature—Glycine, toward More Environmentally Friendly Ionic Forms of Phenoxypropionate-Based Herbicides

Adriana Olejniczak[1], Witold Stachowiak[1], Tomasz Rzemieniecki[1], Michał Niemczak[1,*]

[1] Faculty of Chemical Technology, Poznan University of Technology, Poznan 60-965, Poland

** Corresponding author at: Poznan University of Technology, Berdychowo 4, 61-131 Poznan, Poland; Tel.: +48 616653681. E-mail: michal.niemczak@put.poznan.pl*

The structures of the obtained products were confirmed with the use of ^{1}H and ^{13}C NMR analyses:

Octyldimethylglycine hydrochloride [C_8-Bet][HCl] (3)

^{1}H NMR (CD_3OD) δ [ppm] = 0.90 (t, J = 6.8 Hz, 3H), 1.34 (s, 10H), 1.78 (m, 2H), 3.31 (s, 6H), 3.60 (m, 2H), 4.35 (s, 2H), 11.00 (s, 1H); ^{13}C NMR (CD_3OD) δ [ppm] = 167.3; 66.6; 61.9; 52.1; 32.9; 30.2; 27.3; 23.7; 23.6; 14.5.

Decyldimethylglycine hydrochloride [C_{10}-Bet][HCl] (4)

^{1}H NMR (DMSO-d_6) δ [ppm] = 0.86 (t, J = 6.7 Hz, 3H), 1.24 (s, 14H), 1.67 (m, 2H), 3.22 (s, 6H), 3.52 (m, 2H), 4.42 (s, 2H), 11.01 (s, 1H); ^{13}C NMR (DMSO-d_6) δ [ppm] = 166.2; 64.0; 60.5; 50.5; 31.2; 28.6; 25.7; 22.0; 21.7; 13.9.

Dodecyldimethylglycine hydrochloride [C_{12}-Bet][HCl] (5)

^{1}H NMR (CD_3OD) δ [ppm] = 0.89 (t, J = 6.6 Hz, 3H), 1.29 (s, 18H), 1.78 (m, 2H), 3.31 (s, 6H), 3.60 (m, 2H), 4.34 (s, 2H), 11.03 (s, 1H); ^{13}C NMR (CD_3OD) δ [ppm] = 167.3; 66.5; 61.9; 52.1; 33.1; 30.5; 27.3; 23.7; 23.6; 14.5.

Tetradecyldimethylglycine hydrochloride [C_{14}-Bet][HCl] (6)

^{1}H NMR (CD_3OD) δ [ppm] = 0.89 (t, J = 6,8 Hz, 3H), 1,28 (s, 22H), 1,78 (m, 2H), 3,30 (s, 6H), 3,59 (m, 2H), 4,33 (s, 2H), 11.00 (s, 1H); ^{13}C NMR (CD_3OD) δ [ppm] = 167,3; 66,5; 61,9; 52,1; 33,1; 30,6; 27,3; 25,6; 23,7; 14,5.

Hexadecyldimethylglycine hydrochloride [C_{16}-Bet][HCl] (7)

^{1}H NMR (CD_3OD) δ [ppm] = 0,90 (t, J = 6.9 Hz, 3H), 1.28 (s, 26H), 1.77 (m, 2H), 3.30 (s, 6H), 3,58 (m, 2H), 4.32 (s, 2H), 11.00 (s, 1H); ^{13}C NMR (CD_3OD) δ [ppm] = 167.3; 66.5; 61.9; 52.1; 33.1; 30.7; 27.3; 25.6; 23.7; 14.5.

Butyldimethylglycinium 2-(2,4-dichlorophenoxy)propionate [C_4-Bet][2,4-DP] (8)

^{1}H NMR ($CDCl_3$) δ [ppm] = 0.93 (t, J = 7.3 Hz, 3H), 1.30 (q, J = 7.33 Hz, 2H), 1.62 (d, J = 6.7 Hz, 3H), 1.69 (s, 2H), 3.16 (s, 6H), 3.47 (m, 2H), 3.89 (s, 2H), 4.65 (q, J = 6.7 Hz, 1H), 6.83 (d, J = 8.9 Hz, 1H), 7.11 (dd, $J_{1,3}$ = 8.8 Hz, $J_{1,2}$ = 2.5 Hz, 1H), 7.32 (d, J = 2.5 Hz, 1H), 10.95 (s, 1H); ^{13}C NMR ($CDCl_3$) δ [ppm] = 174.2; 166.1; 152.4; 129.6; 127.4; 125.7; 123.6; 115.4; 74.4; 64.4; 63.9; 51.0; 24.4; 19.4; 18.4; 13.3.

Hexyldimethylglycinium 2-(2,4-dichlorophenoxy)propionate [C_6-Bet][2,4-DP] (9)

^{1}H NMR ($CDCl_3$) δ [ppm] = 0.88 (t, *J* = 6.7 Hz, 3H), 1.27 (s, 6H), 1.62 (d, *J* = 6.7 Hz, 3H), 2.17 (s, 2H), 3.14 (s, 6H), 3.44 (m, 2H), 3.88 (s, 2H), 4.64 (q, *J* = 6.8 Hz, 1H), 6.83 (d, *J* = 8.8 Hz, 1H), 7.10 (dd, $J_{1,3}$ = 8.8 Hz, $J_{1,2}$ = 2.6 Hz, 1H), 7.32 (d, *J* = 2.5 Hz, 1H), 10.95 (s, 1H); ^{13}C NMR ($CDCl_3$) δ [ppm] = 174.3; 166.4; 152.4; 129.6; 127.1; 125.7; 123.5; 115.4; 74.5; 64.6; 64.0; 50.9; 31.0; 25.7; 22.5; 22.2; 18.4; 13.7.

Decyldimethylglycinium 2-(2,4-dichlorophenoxy)propionate [C_{10}-Bet][2,4-DP] (11)

^{1}H NMR ($CDCl_3$) δ [ppm] = 0.88 (t, *J* = 6.9 Hz, 3H), 1.25 (s, 14H), 1.62 (d, *J* = 6.8 Hz, 3H), 2.09 (s, 2H), 3.13 (s, 6H), 3.43 (m, 2H), 3.88 (s, 2H), 4.65 (q, *J* = 6.8 Hz, 1H), 6.83 (d, *J* = 8.8 Hz, 1H), 7.11 (dd, $J_{1,3}$ = 8.7 Hz, $J_{1,2}$ = 2.5 Hz, 1H), 7.33 (d, *J* = 2.5 Hz, 1H), 11.00 (s, 1H); ^{13}C NMR ($CDCl_3$) δ [ppm] = 174.1; 166.6; 152.4; 129.7; 127.4; 125.8; 123.7; 115.4; 74.3; 64.7; 64.0; 50.9; 31.7; 29.3; 29.0; 26.1; 22.5; 18.4; 14.0.

Dodecyldimethylglycinium 2-(2,4-dichlorophenoxy)propionate [C_{12}-Bet][2,4-DP] (12)

^{1}H NMR ($CDCl_3$) δ [ppm] = 0.88 (t, *J* = 6.8 Hz, 3H), 1.26 (s, 18H), 1.63 (d, *J* = 6.7 Hz, 3H), 2.17 (s, 2H), 3.17 (s, 6H), 3.45 (m, 2H), 3.89 (s, 2H), 4.65 (q, *J* = 6.8 Hz, 1H), 6.84 (d, *J* = 8.9 Hz, 1H), 7.10 (dd, $J_{1,3}$ = 8.8 Hz, $J_{1,2}$ = 2.6 Hz, 1H), 7.32 (d, *J* = 2.5 Hz, 1H), 11.00 (s, 1H); ^{13}C NMR ($CDCl_3$) δ [ppm] = 174.4; 166.3; 152.5; 129.6; 127.3; 125.7; 123.6; 115.4; 74.5; 64.6; 64.0; 51.0; 31.7; 29.5; 26.1; 24.0; 22.5; 18.4; 13.9.

Hexadecyldimethylglycinium 2-(2,4-dichlorophenoxy)propionate [C_{16}-Bet][2,4-DP] (14)

^{1}H NMR ($CDCl_3$) δ [ppm] = 0.88 (t, *J* = 6.7 Hz, 3H), 1.26 (s, 26H), 1.64 (d, *J* = 6.9 Hz, 3H), 2.69 (s, 2H), 3.17 (s, 6H), 3.47 (m, 2H), 3.90 (s, 2H), 4.64 (d, *J* = 6.8 Hz, 1H), 6.83 (d, *J* = 8.9 Hz, 1H), 7.11 (dd, $J_{1,3}$ = 8.8 Hz, $J_{1,2}$ = 2.6 Hz, 1H), 7.32 (d, *J* = 2.6 Hz, 1H), 11.00 (s, 1H); ^{13}C NMR ($CDCl_3$) δ [ppm] =174.6; 166.5; 152.6; 129.7; 127.3; 125.7; 123.7; 115.4; 74.7; 64.7; 64.1; 51.0; 31.7; 29.5; 29.0; 26.1; 22.5; 18.5; 14.0.

Table S1. Chemical shifts [ppm] of selected protons in ^{1}H NMR spectra of products (**3**–**7**).

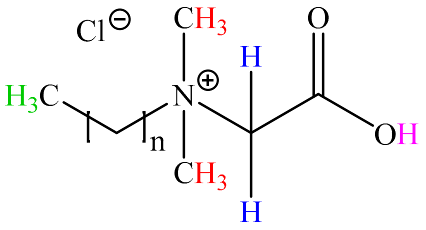

No	n	CH_3	N-(CH_3)$_2$	N-CH_2-CO	COOH
**3**[a]	7	0.90(t)	3.31(s)	4.35(s)	11.00(s)
**4**[b]	9	0.86(t)	3.22(s)	4.42(s)	11.01(s)
**5**[a]	11	0.89(t)	3.31(s)	4.34(s)	11.03(s)
**6**[a]	13	0.89(t)	3.30(s)	4.33(s)	11.00(s)
**7**[a]	15	0.90(t)	3.30(s)	4.32(s)	11.01(s)

s – singlet, t – triplet, solvent - [a] CD_3OD, [b] DMSO-d_6

Table S2. Chemical shifts [ppm] of selected carbons in ^{13}C NMR spectra of products (**3**–**7**).

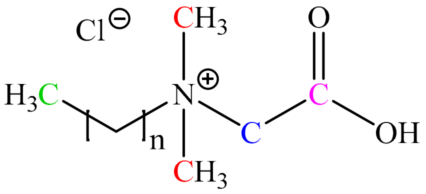

No	n	CH_3	N-(CH_3)$_2$	N-CH_2-CO	COOH
**3**[a]	7	14.5	52.1	61.9	167.3
**4**[b]	9	13.9	50.5	60.5	166.3
**5**[a]	11	14.5	52.1	61.9	167.3
**6**[a]	13	14.5	52.1	61.9	167.3
**7**[a]	15	14.5	52.1	61.9	167.3

solvent - [a] CD_3OD, [b] DMSO-d_6

Table S3. Chemical shifts [ppm] of selected protons from cation in ^{1}H NMR spectra of ILs.

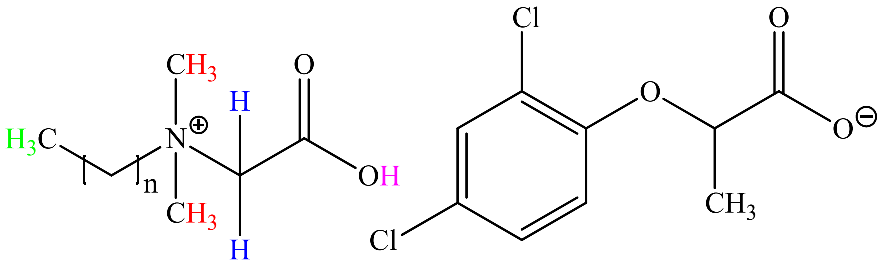

No	n	CH_3-R	N-$(CH_3)_2$	N-CH_2-CO	COOH
**8**	7	0.93(t)	3.16(s)	3.89(s)	10.95(s)
**9**	9	0.88(t)	3.14(s)	3.88(s)	10.95(s)
**11**	11	0.88(t)	3.13(s)	3.88(s)	11.00(s)
**12**	13	0.88(t)	3.17(s)	3.89(s)	11.00(s)
**14**	15	0.88(t)	3.17(s)	3.90(s)	11.01(s)

s – singlet, t – triplet, solvent – $CDCl_3$

Table S4. Chemical shifts [ppm] of selected protons from anion in ^{1}H NMR spectra of ILs.

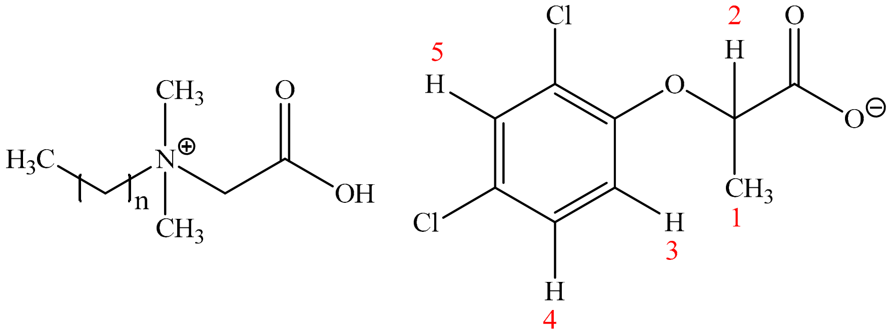

No	n	1	2	3	4	5
**8**	7	1.62(d)	4.64(q)	6.83(d)	7.11(dd)	7.32(d)
**9**	9	1.62(d)	4.64(q)	6.83(d)	7.10(dd)	7.32(d)
**11**	11	1.62(d)	4.65(q)	6.83(d)	7.11(dd)	7.33(d)
**12**	13	1.63(d)	4.65(q)	6.84(d)	7.10(dd)	7.32(d)
**14**	15	1.64(d)	4.64(d)	6.83(d)	7.11(dd)	7.32(d)

d – doublet; dd – doublet of doublets, q – quartet, solvent – $CDCl_3$

Table S5. Chemical shifts [ppm] of selected carbons from cation in ^{13}C NMR spectra of ILs.

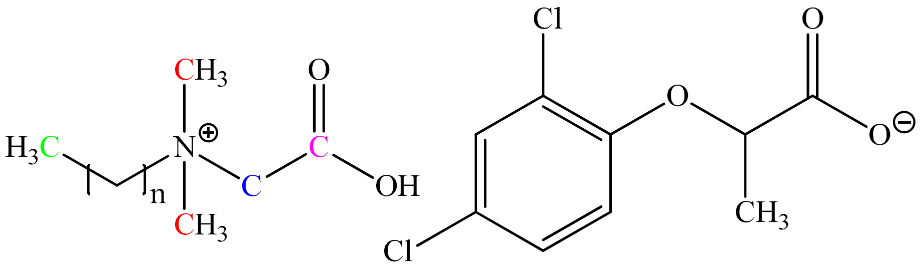

No	n	CH_3-R	N-$(CH_3)_2$	N-CH_2-CO	COOH
**8**	7	13.3	51.0	63.9	166.6
**9**	9	13.7	50.9	64.0	166.4
**11**	11	14.0	50.9	64.0	166.6
**12**	13	13.9	51.0	64.0	166.3
**14**	15	14.0	51.0	64.1	165.5

solvent – $CDCl_3$

Table S6. Chemical shifts [ppm] of selected carbons from anion in ^{13}C NMR spectra of ILs.

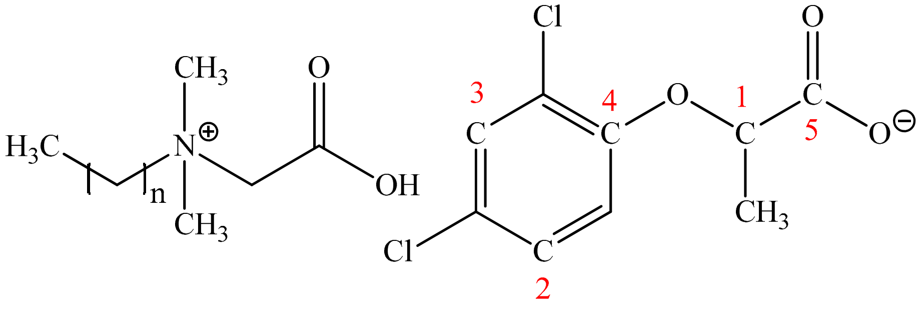

No	n	1	2	3	4	5
**8**	7	74.4	127.4	129.6	152.4	174.2
**9**	9	74.5	127.4	129.6	152.4	174.3
**11**	11	74.3	127.4	129.7	152.4	174.1
**12**	13	74.5	127.3	129.6	152.5	174.4
**14**	15	74.7	127.3	129.7	152.6	174.6

solvent – $CDCl_3$

Table S7. Densities [g·cm^{-3}] of ILs **8–14** in a temperature range from 20 to 80 °C.

**No**	**Temperature [ºC]**						
	**20**	**30**	**40**	**50**	**60**	**70**	**80**
**8**	1.23	1.22	1.21	1.21	1.20	1.19	1.18
**9**	1.21	1.20	1.19	1.18	1.18	1.17	1.16
**10**	1.16	1.15	1.14	1.13	1.13	1.12	1.11
**11**	1.15	1.14	1.13	1.12	1.11	1.10	1.10
**12**	1.13	1.12	1.11	1.10	1.09	1.09	1.08
**13**	1.08	1.07	1.07	1.06	1.05	1.04	1.04
**14**	1.08	1.07	1.06	1.05	1.05	1.04	1.03

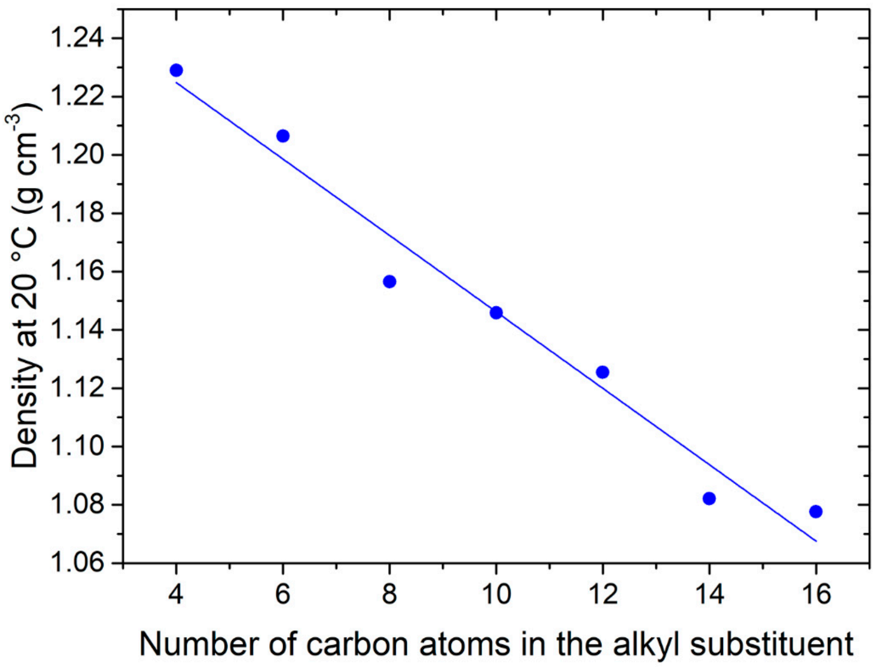

Figure S1. Relationship between alkyl substituent length in the cation and the densities of ILs **8–14** at 20 °C.

Table S8. Refractive indices of ILs **8–14** in a temperature range from 20 to 80 °C.

No	Temperature [ºC]						
	**20**	**30**	**40**	**50**	**60**	**70**	**80**
**8**	1.522	1.517	1.513	1.509	1.506	1.503	1.499
**9**	1.521	1.517	1.514	1.511	1.508	1.505	1.502
**10**	1.508	1.504	1.501	1.497	1.494	1.492	1.490
**11**	1.507	1.504	1.500	1.497	1.494	1.491	1.489
**12**	1.503	1.500	1.496	1.493	1.492	1.489	1.488
**13**	1.497	1.494	1.491	1.488	1.486	1.485	1.482
**14**	1.498	1.493	1.490	1.486	1.484	1.482	1.479

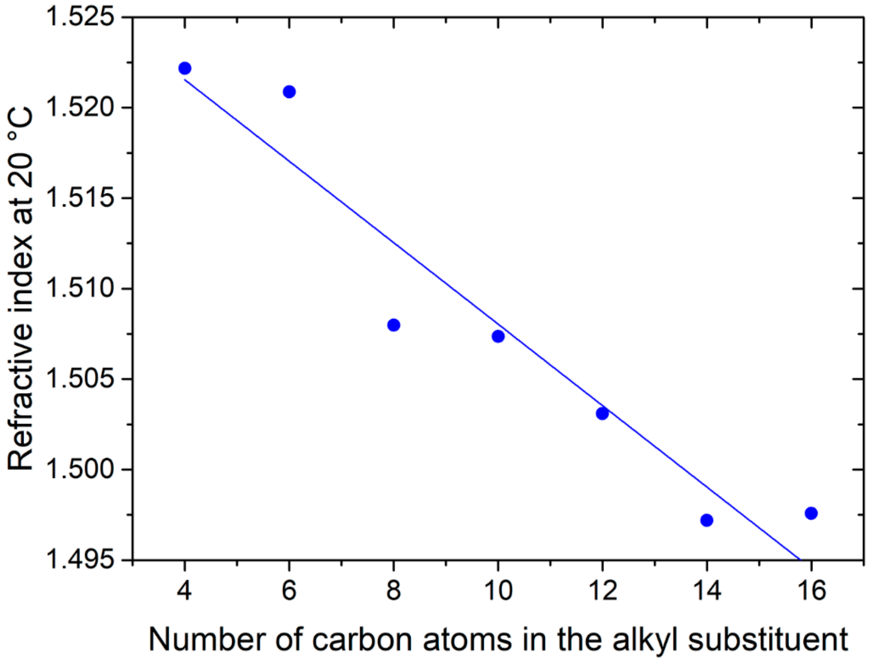

Figure S2. Relationship between alkyl substituent length in the cation and the refractive indices of ILs **8–14** at 20 °C.

Table S9. Viscosities [Pa·s] of ILs **8–14** in a temperature range from 20 to 80 °C.

No	Temperature [ºC]						
	**20**	**30**	**40**	**50**	**60**	**70**	**80**
**8**	480.73	81.78	18.14	5.27	1.78	0.71	0.31
**9**	1725.30	257.93	53.04	14.25	4.70	1.85	0.80
**10**	1800.70	282.17	61.22	17.15	5.79	2.30	1.01
**11**	54.35	15.25	5.14	2.06	0.92	0.46	0.26
**12**	48.50	14.41	5.14	2.12	0.98	0.50	0.29
**13**	14.15	4.88	1.94	0.88	0.44	0.25	0.15
**14**	38.70	11.34	4.04	1.65	0.76	0.39	0.22

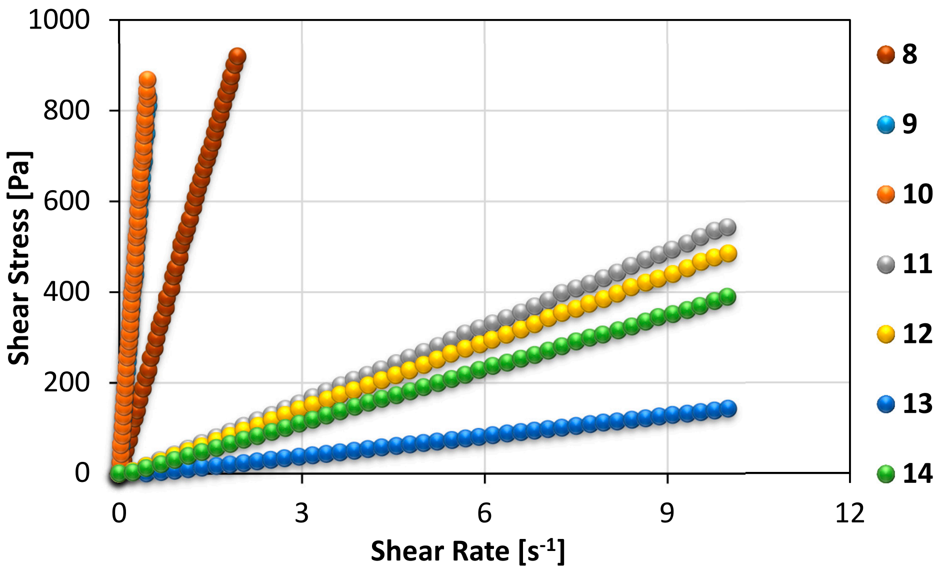

Figure S3. Shear stress versus shear rate for ILs **8–14.**

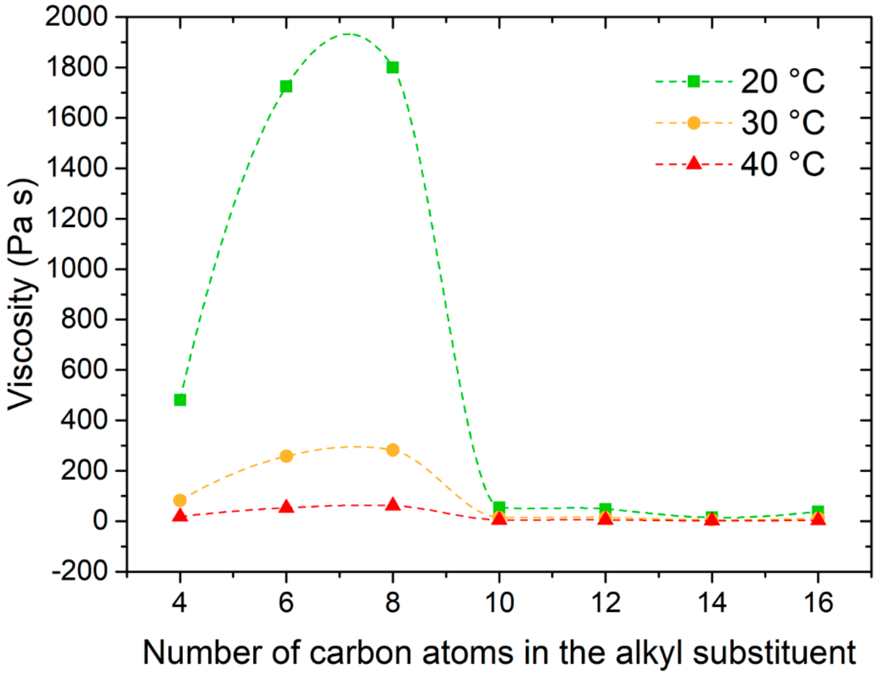

Figure S4. Relationship between alkyl substituent length in the cation and the viscosities of ILs **8–14** at 20, 30 and 40 °C.

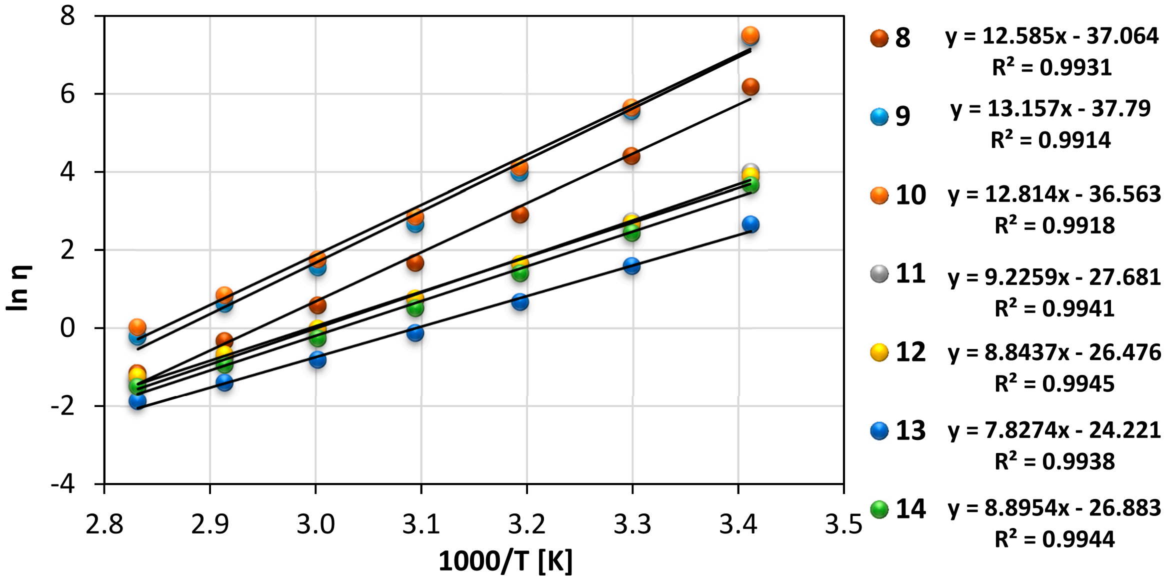

Figure S5. Arrhenius plots for ILs **8–14**, where η is viscosity and T is temperature.

Table S10. Biological activity of ILs **8–14** toward oilseed rape; **REF1** – potassium 2-(2,4-dichlorophenoxy)propionate, **REF2** – Aminopielik Standard 600 SL.

**Substance***	**R**	**SE** [%]	**Mass reduction** [%]
**Untreated control**	-	2.5	0 a***
**8**	C_4H_9	4.9	16.8 b
**9**	C_6H_{13}	4.3	19.0 b
**10**	C_8H_{17}	2.1	44.7 cd
**11**	$C_{10}H_{21}$	7.2	46.1 cd
**12**	$C_{12}H_{25}$	3.5	55.5 d
**13**	$C_{14}H_{29}$	3.7	50.9 cd
**14**	$C_{16}H_{33}$	6.9	45.6 cd
**REF1**	-	2.8	42.4 c
**REF2**	-	3.7	11.3 b
HSD (0.05)			10.9

*All tested products were used in a dose of 400 g of active ingredient per hectare

** **SE** – standard error

*** a–d different letters indicate statistically different mean HSD ($p < 0.05$)

Table S11. Green Chemistry Metrics. [1]

No	*Atom economy* (%)	*Percentage yield* (%)	*Reaction mass efficiency*	*Environmental factor** (E-factor)
**8**	72	74	0.54	0.43
**9**	73	63	0.47	0.44
**10**	75	72	0.54	0.38
**11**	76	84	0.64	0.33
**12**	77	81	0.63	0.32
**13**	78	81	0.64	0.30
**14**	79	83	0.66	0.28

* Higher E-factors are relatively less desirable. For bulk chemicals, manufactured in amounts of hundreds of thousands to millions of tons per year, tolerable E-factors typically range from 1 to 5. For commercially produced pharmaceuticals E-factor is extremely high and generally exceeds 50. The elaborated methodology exhibits E-factor lower than 1, hence, can be considered as sustainable and appropriately developed in accordance to green chemistry principles.

[1] Sheldon, R.A. Metrics of Green Chemistry and Sustainability: Past, Present, and Future. *ACS Sustainable Chem. Eng.* **2018**, *6,* 32−48, doi:10.1021/acssuschemeng.7b03505.
